# Cellulose Nanocrystal Isolation from Hardwood Pulp using Various Hydrolysis Conditions

**DOI:** 10.3390/molecules24203724

**Published:** 2019-10-16

**Authors:** Kuan-Hsuan Lin, Toshiharu Enomae, Feng-Cheng Chang

**Affiliations:** 1 Graduate School of Life and Environmental Science, University of Tsukuba, Tsukuba 305-8572, Japan; apfel5061@gmail.com (K.-H.L.); t@enomae.com (T.E.); 2 School of Forestry and Resources Conservation, National Taiwan University, Taipei 10617, Taiwan; 3 Advanced Research Center for Green Materials Science and Technology, National Taiwan University, Taipei 10617, Taiwan

**Keywords:** cellulose nanocrystals, hardwood pulp, sulfuric acid hydrolysis

## Abstract

To expand the application field of the pulping industry, this study conducted a series of sample preparations for processing cellulose nanocrystals (CNCs) from a dry hardwood pulp to achieve optimal sulfuric acid hydrolysis. The properties of laboratory-prepared pulp CNCs (P-CNCs) were investigated with different preparation conditions including sulfuric acid concentrations, hydrolysis temperatures, and hydrolysis durations. Results showed a gradient of color changes observed with the increase of hydrolysis duration and temperature. Under certain conditions, the derived P-CNCs exhibited nanoscale dimensions, detected by transmission electron microscopy, and a crystallinity index similar to commercial products. In addition, the surface sulfate groups were assumed to be contributed by sulfuric acid hydrolysis. However, a high acid concentration and long hydrolysis processing duration introduced more sulfate groups on the derived P-CNCs, which may have acted as flame retardants and, thus, increased the amount of char residue.

## 1. Introduction

Cellulose is a sustainable bioresource and is abundant in nature. Because of the unique characteristics of cellulose, cellulosic materials have recently gained considerable global attention in the field of materials science, particularly in exploring and applying such materials for industrial use. Because of the hydroxyl groups, intra- and inter-molecular hydrogen bonds are formed, and cellulose chains are arranged in a highly ordered crystalline region, whereas the remaining disordered structure is called an amorphous region. With the nanometric size effect, the crystalline regions, extracted to be cellulose nanocrystals (CNCs), have recently been studied in the development of biocomposites [1]. For the design of a CNC composite, CNCs have the potential to serve as a functional reinforcement material, providing enhanced thermal, mechanical, optical, and gas-barrier properties, as a result of their highly ordered nanostructure [2].

Owing to the steric effect, the hydroxyl groups on cellulose chains exhibit different reactivity characteristics. With the numbering system of carbon atoms, the locations of hydroxyl groups are determined in an anhydroglucose unit of cellulose. Thus, hydroxyl groups on the surface of cellulose can react with various chemicals and become derivatives with designed functions. However, the CNC extraction procedure plays an essential role in modifying the surface chemistry of the particles; thus, ideal hydrolysis conditions are important for consistently producing high-quality CNC products.

Chemical hydrolysis, particularly acid hydrolysis, has been proven as an effective solution for generating highly pure CNCs [1,3,4,5]. The reviewed literature suggests that preparation methods introduce different chemical groups onto the surface of CNCs through various acids [3,6,7,8]. Phosphoric acid hydrolyzed samples showed a low surface charge [3]. Besides, hydrochloric acid hydrolyzed samples exhibited more intermolecular hydrogen bonding [8]. In this study, sulfuric acid was chosen to produce products with a high aspect ratio and with a negatively charged surface so that the resulting CNCs were expected to disperse in an aqueous system due to electrostatic repulsion between the particles [9,10,11]. However, dried CNC powder is preferred because of its easy delivery, preference for further analysis, and antibacterial and antifungal properties, though it has been reported to be difficult to disperse in water [12]. To reach a better re-dispersibility, the recommended counterion exchange for neutral monovalent cations [13] was tried in this study.

The crystallinity was reported to be dependent for the reason that bacterial and tunicate CNCs provided a higher crystalline fraction than wood CNCs, or the pure cellulose CNCs provided a crystallinity index (*Cr.I.*) higher than wastepaper CNCs [14,15]. However, pretreatment of cellulose materials before acid hydrolysis also played a role in influencing the *Cr.I.* value, such as pretreatments with alkali extraction and bleaching [16]. Thus, with careful and proper pretreatment, even a kraft industry by-product was proven to reach high CNC crystallinity [11]. Besides crystallinity, the raw material was also reported to have a different length. Waste paper or a softwood pulp was treated with a sulfuric acid hydrolysis process, and products with a length of around 200 nm were produced [15,17,18]. Other than the influence of raw material, it was reported that, generally, CNCs with higher surface charges and narrower sizes would result in products with higher acid concentrations and hydrolysis temperatures as well as longer hydrolysis durations [1,17,18,19,20]. On the other hand, the relationship between yield, sulfuric acid concentration, hydrolysis temperature, and hydrolysis duration was investigated with softwood sulfite pulp, which affected the sulfate group density of CNCs [10]. Consequently, the optimal condition of such material is expected to be clarified on a one-by-one basis when it comes to each source. The relationship between the hydrolysis condition and the resulting morphology of CNCs is worth studying.

To the best of our knowledge, consistent production of high-quality CNCs from pulp, as well as recycled pulp, is still difficult because of the complex chemical conditions. With the purpose to expand the possibilities in the pulp and paper-making industry, we intended to clarify and justify the factors of the preparation conditions in considering CNC properties with hardwood pulp raw material. A series of sulfuric acid hydrolyses was selected to extract pure CNCs from hardwood pulp with the objective of studying the formation of negatively charged sulfate groups, and processing conditions were investigated to assess the factors affecting the hydrolyzed CNCs. Moreover, several properties, such as morphology, surface properties, crystallinity, and thermal stability, were tested and measured to pursue an optimal procedure for CNC isolation from hardwood pulp.

## 2. Materials and Methods

### 2.1. Materials

The bleached, dry hardwood pulp applied here (provided by Chung Hwa Pulp Corporation, Taiwan) was a mixture of eucalyptus (Eucalyptus spp.) and bixa (Bixa spp.). The chemical composition of the applied dry hardwood pulp was mainly cellulose (85–89%) and hemicelluloses (8–10%) with small impurity levels of lignin (<1%), ash (<0.5%), and lipid (<0.5%). Sodium hydroxide (Showa Chemical Co. Ltd., Japan) was used for pretreatment and neutralization after acid hydrolysis. Sulfuric acid (95–98%, *w*/*w*) (Scharlau, Barcelona [21], Spain) was prepared into various concentrations for hydrolysis. The water used for this purpose was double-distilled water (DD water).

### 2.2. CNCs Preparation

**Figure 1:**
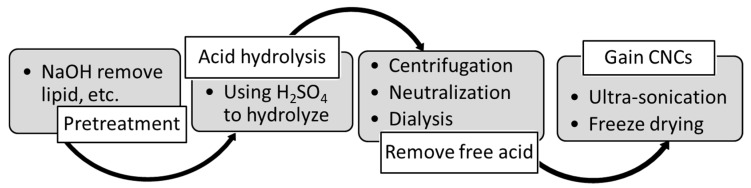
The procedure of CNC preparation.

The bleached hardwood pulp was first to cut into strips, after which the following steps were taken (Figure 1). Though the pulp was already bleached, alkaline extraction was performed as a pretreatment for the dry hardwood pulps. Strips of pulp were treated with 3% (*w*/*w*) NaOH at 50 °C for 2 h, which would remove fatty acids, residual lignin, hemicelluloses, resin, and other impurities. Simultaneously, the amorphous region would also swell so that it would improve the effectiveness of acid hydrolysis later.

Pretreatment was followed by a second step: acid hydrolysis. Products gained from the first step were filtered by using an aspirator (A100-S, EYELA, Tokyo, Japan) and a glass filter. Then, the pulp was hydrolyzed in air in a variety of conditions, as shown in Table 1, including sulfuric acid at concentrations of 46% and 63% (*w*/*w*), hydrolysis temperatures of 45 and 65 °C, and hydrolysis durations of 0.5, 1, and 2 h (Table 1). The weight ratio of hardwood pulp to acid solution was set to 1:20 (g/mL), and P-CNCs were obtained after acid hydrolysis. After hydrolysis, 300 mL of DD water was added to stop the reaction. Then, a third step was conducted to remove the free acid. The suspension was transferred to a 50 mL centrifuge tube and then centrifuged at 5000 rpm for 10 min at least 3 times. Finally, P-CNCs were dried to a powder in the fourth step: ultrasound treatment. In addition, samples denoted “200P” were prepared from pre-milled pulp powder, with a hydrodynamic size of approximately 200 nm, to evaluate the case of recycled short-fibers. The 200P samples were also prepared using the 4-step procedure as illustrated above.

Titration was then conducted with a 10% (*w*/*w*) NaOH solution to gain redispersible P-CNC suspensions before dialyzing against water through membranes with a cutoff molecular weight of 12,000–14,000 (Membrane Filtration Products Inc., Seguin, TX, USA). Freeze-drying was chosen in this study because it has been reported as a way to dry samples without forming significantly larger aggregates than those seen in air-drying or spray-drying [14]. Thus, P-CNC powders were obtained by freeze-drying (Kingmech Co. Ltd., Taipei, Taiwan) after ultrasonication for 20 min (Crest Ultrasonics Corporation, Ewing Township, NJ, USA).

Finally, a relationship between the resulting properties and the hydrolysis conditions was observed. Commercially available CNCs (sample C) (CNC—Cellulose Nanocrystals Freeze-dried, University of Maine, Orono, ME, USA) were analyzed and set as the reference standard for evaluation of laboratory-made P-CNCs. The dimension, surface morphology, crystallinity index, and thermal stability were measured to determine the relationship between the manufacturing process conditions and resulting properties.

### 2.3. CNC Product Evaluation

#### 2.3.1. Product Size

CNC suspensions at 0.1% (*w*/*w*) were dropped on 20-mesh carbon-coated copper grids for transmission electron microscopy (TEM) observation without any treatment for contrast. The samples were observed using TEM (JEM-1200 EXII, JEOL and H7650, Hitachi, Tokyo, Japan) at 60 kV. Dimensions of at least 30 fibers were analyzed using *ImageJ* (https://imagej.nih.gov/ij/index.html). Only isolated particles with clear edges were measured, and the width at the center of each particle was determined as the diameter.

#### 2.3.2. Crystallinity

**Figure 2:**
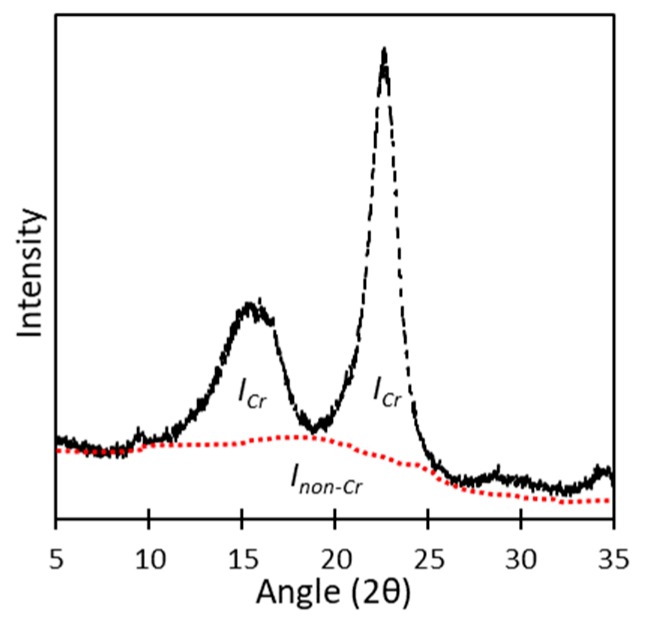
Calculation of crystallinity index. Take the sample “SA6345-2PN” as example.

Regarding the configuration of crystalline regions, wide-angle X-ray diffraction (WAXD; X’Pert Pro, Malvern Panalytical Ltd., Royston, United Kingdom) was used to evaluate the crystallinity index (*Cr.I.*) of the CNCs. The Ruland–Vonk X-ray diffraction method [21], similar to the “amorphous subtraction method” employed in another study [22], was applied to calculate *Cr.I.* from the ratio of the crystalline phase area to the total area including the non-crystalline phase (Figure 2 and Equation (1)). In Equation (1), *I_Cr_* is the integrated intensity of the crystalline phase, and *I_non-Cr_* is the integrated intensity of the non-crystalline phase as a background.

(1) $$Cr.I.\;\left(\%\right)=\frac{I_{Cr}}{I_{Cr}+I_{non-Cr}}\times 100\left(\%\right)$$

#### 2.3.3. Thermal Stability

A thermal gravimetric analyzer (TGA; TGA/SDTA 851, METTLER, Columbus, OH, USA) was applied to observe the degradation behavior of CNCs under heating. A degradation diagram was collected through placing approximately 2 mg of CNCs into an alumina pan and heating from 80 to 800 °C at a heating rate of 20 °C/min under nitrogen. Then, we defined the degradation temperature as the point of a zero crossing the second derivate of the thermal degradation curve.

#### 2.3.4. Surface Chemistry Information

An electron probe micro analyzer (EPMA; JXA-8530F, JEOL, Tokyo, Japan) was applied to investigate the elemental composition at the surface of CNCs. Samples of P-CNCs were coated with carbon and measured at an accelerating voltage of 10 kV. On the other hand, diffuse reflectance infrared Fourier transform spectroscopy (FT-IR; FTS-40, BIO-RAD, Hercules, CA, USA) and X-ray Photoelectron Spectroscopy (XPS; JPS-9010TR, JEOL, Tokyo, Japan) were applied to detect the surface functional groups and chemical bonds of CNCs.

## 3. Results and Discussions

### 3.1. The Outward Appearance and Product Size

**Figure 3:**
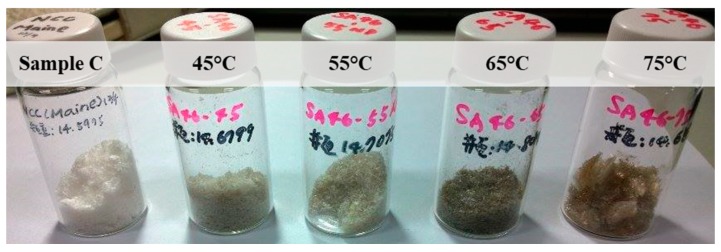
P-CNCs prepared at different hydrolysis temperatures—45, 55, 65, and 75 °C from left to right—compared with the standard (sample C).

Owing to excessive hydrolysis, oligomers or levoglucosans were formed in the cases of strong hydrolysis conditions. The color obviously changed from white to dark brown as the hydrolysis temperature increased from 45 °C to 75 °C (Figure 3). Compared to the reference sample C, P-CNCs hydrolyzed at 45 °C exhibited a white color more akin to sample C than the other samples.

**Figure 4:**
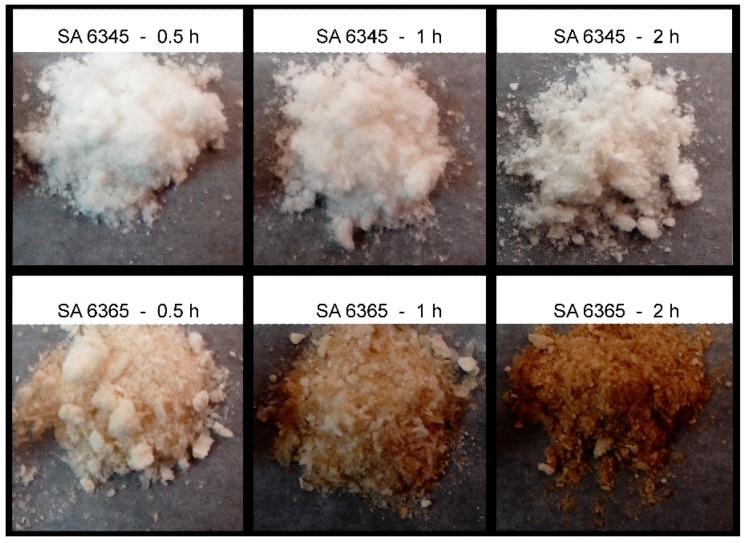
CNCs prepared via different conditions.

In addition, more significant color changes were found as the hydrolysis duration increased. Samples treated at a temperature as high as 65 °C (Figure 4, bottom panels) exhibited a more obvious color change than those treated at a lower temperature (45 °C; Figure 4, upper panels), as the hydrolysis duration increased from 0.5 to 1 to 2 from left to right in Figure 4.

**Figure 5:**
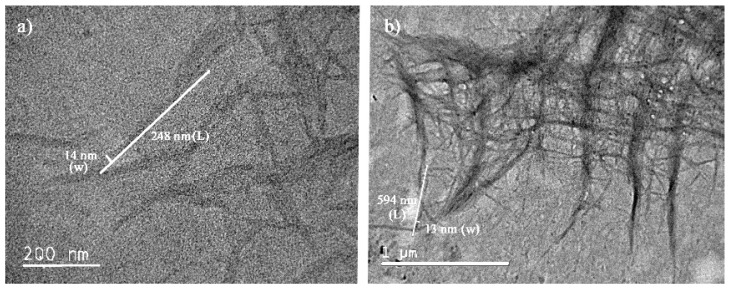
TEM observation of P-CNCs prepared with different sulfuric acid concentrations: (**a**) 63%; (**b**) 46%.

In contrast to the width, a significant difference of length between samples was noticed. Samples prepared using a high sulfuric acid concentration (63%) were shorter in length than those obtained using a low concentration (46%). The preferred length of CNCs is shorter than 500 nm; therefore, a sulfuric acid concentration as high as 63% (*w*/*w*) was suggested for use in the P-CNC preparation procedure (Figure 5).

**Figure 6:**
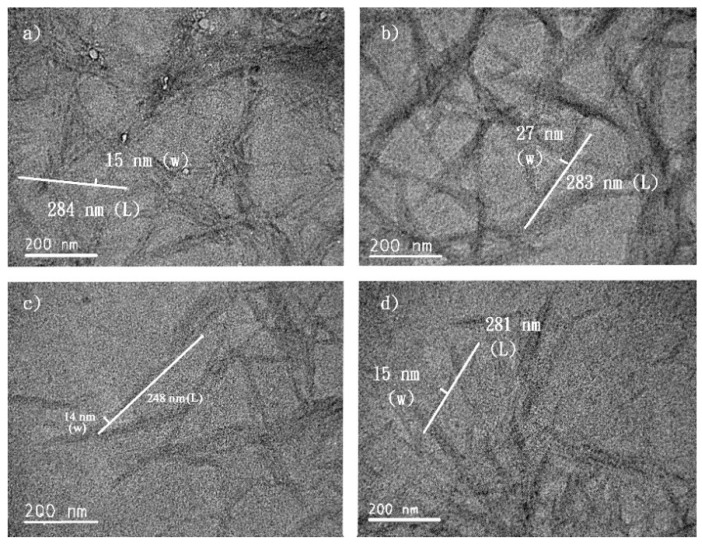
P-CNCs prepared with 63% sulfuric acid at 45 °C for (**a**) 0.5 h, (**b**) 1 h, and (**c**) 2 h and at 65 °C for (**d**) 2 h.

**Figure 7:**
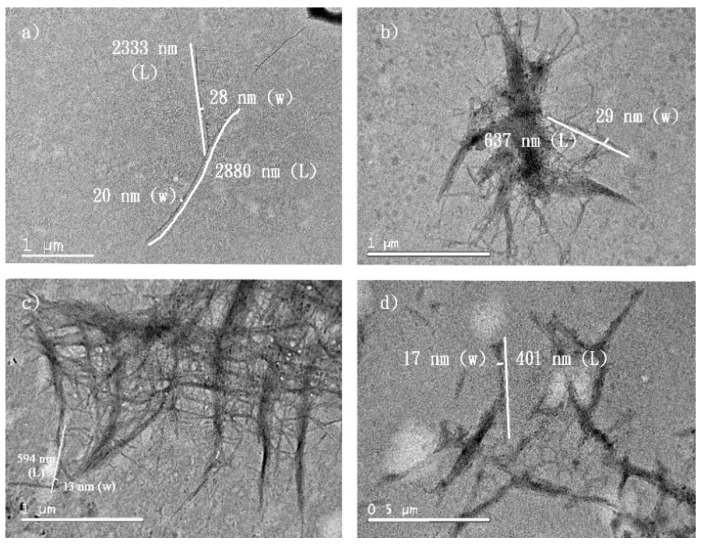
P-CNCs prepared with 46% sulfuric acid at 45 °C for (**a**) 0.5 h, (**b**) 1 h, and (**c**) 2 h and at 65 °C for (**d**) 2 h.

At a high sulfuric acid concentration, the hydrolysis temperature and duration did not significantly affect the physical appearance of P-CNCs. Rod- or needle-shaped P-CNCs of similar dimensions were clearly observed through TEM images (Figure 6). However, when the concentration of sulfuric acid was as low as 46% (*w*/*w*), the hydrolysis temperature and duration influenced the length of the observed P-CNCs, which might not achieve complete hydrolysis to small-crystallized particles. P-CNCs obtained from hydrolysis for 0.5 h were longer than those hydrolyzed for 2 h; they were shorter after being treated at 65 °C compared with those treated at 45 °C (Figure 7).

**Figure 8:**
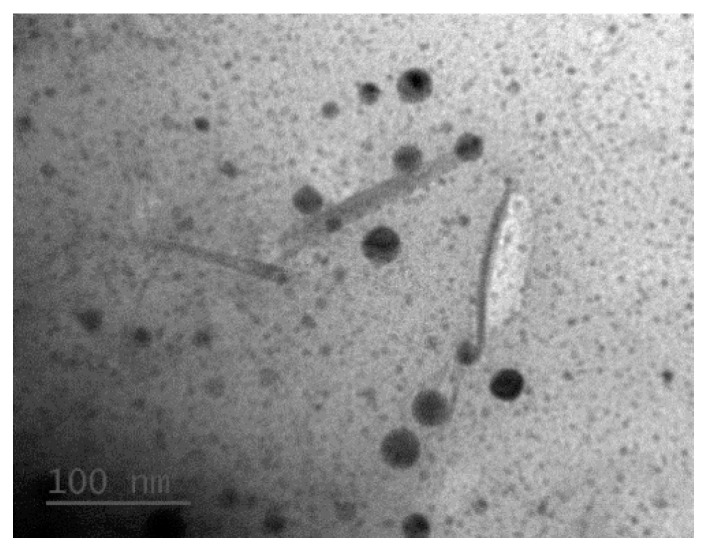
TEM observation of sample “200P”.

The results of the 200P samples indicated an average length of approximately 100 nm, as determined through TEM measurements (Figure 8). However, numerous unknown impurities (black spots in Figure 8) were observed under each hydrolysis condition of 200P. Further analyses of such conditions would result in a low yield (approximately 20%). Also, because of the high cost of ball milling, the method of pre-milling raw materials to approximately 200 nm was not recommended to prepare CNCs.

### 3.2. Crystallinity

**Figure 9:**
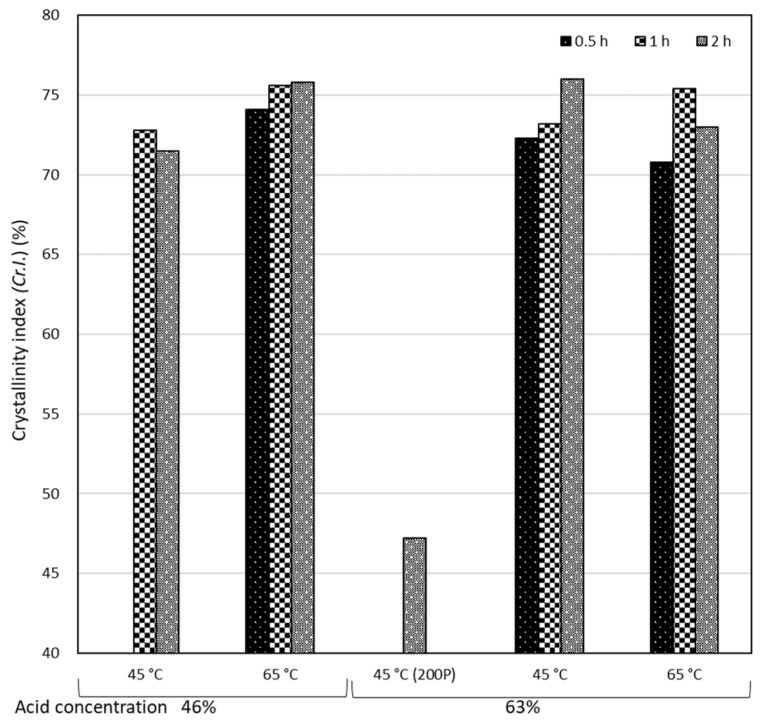
Crystallinity index of CNCs prepared in different conditions.

The crystallinity index (*Cr.I.*) was calculated from WAXD patterns through the Ruland–Vonk X-ray diffraction method and shown in Figure 9. The average *Cr.I.* value of 200P was approximately 50%. The pre-milling treatment was assumed to be the reason for such a low *Cr.I.* value because milling can decrease the crystallite size and destroy crystalline regions. All other samples exhibited an average *Cr.I.* value of approximately 70–80%, which was close to sample C (76%). Moreover, samples prepared using a longer hydrolysis duration tended to exhibit higher *Cr.I.* values than those prepared for a shorter duration (2 h ≥ 1 h > 0.5 h, or 1 h ≥ 2 h > 0.5 h), suggesting that the longest hydrolysis duration of nearly 1–2 h would be the most suitable condition among the three durations to produce P-CNCs with a high crystallinity index.

### 3.3. Thermal Stability

**Figure 10:**
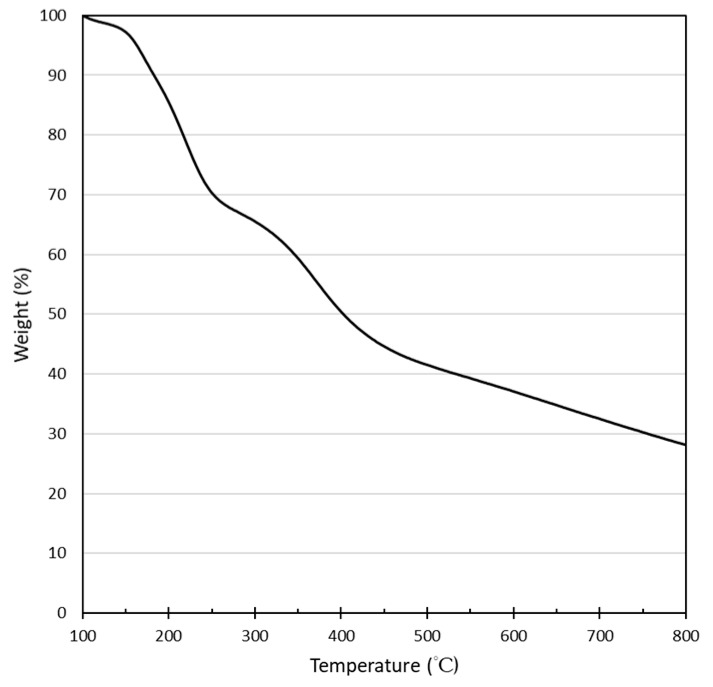
TGA diagram of sample “200P”.

The TGA diagram of 200P revealed a two-step degradation process, as illustrated in Figure 10. The first degradation period started at around 140 °C and then entered a short, stable stage when nearly 65% of the CNCs in weight were degraded at around 240 °C, after which the second degradation period started at around 270 °C.

A previous study reported that the decomposition temperature of glucose started at around 158 °C [23], and the thermal degradation reaction had been pointed out to be catalyzed by sulfate groups. The first degradation period of 200P started at around 150 °C [24]. Then, the crystal interior was degraded in a second degradation process at around 300 °C [24].

**Figure 11:**
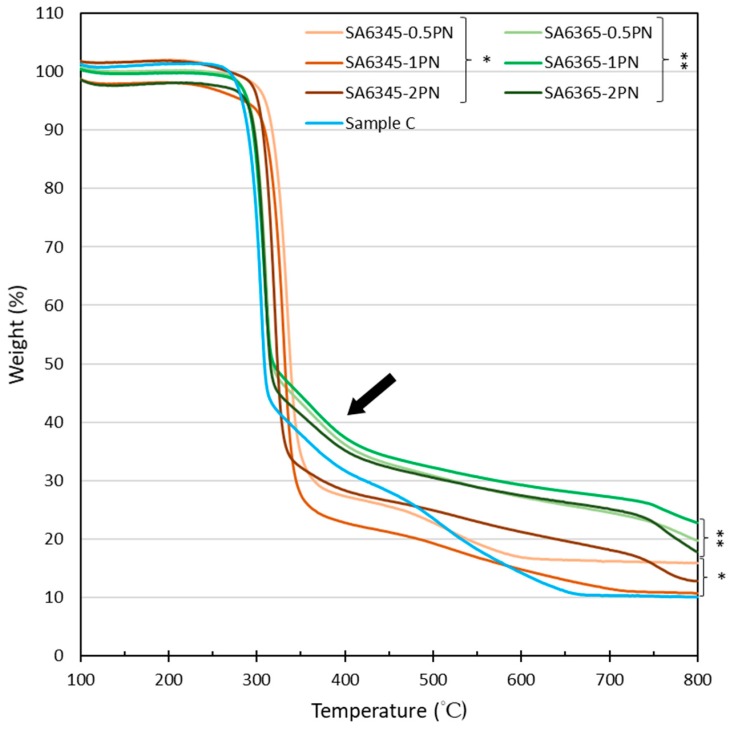
TGA diagram of all samples: P-CNCs obtained through hydrolysis at 65 °C (marked as “**”) and 45 °C (marked as “*”).

In the series of 63% sulfuric acid hydrolysis in P-CNCs (Figure 11), all the samples exhibited a degradation temperature close to reference sample C (blue line). Unlike “200P”, the samples exhibited an onset temperature of main degradation higher than 270 °C, and crystalline regions contributed to degradation temperatures higher than 300 °C. However, severe hydrolysis conditions, such as high temperature and long duration, were assumed to introduce more sulfate groups on P-CNCs. A lower degradation temperature (onset temperature as well as the main degradation temperature) was noticed in the case of P-CNCs obtained through hydrolysis at 65 °C (marked as “**” in Figure 11), which suggested that the sulfate groups had a negative effect on thermal stability. On the other hand, with the assumed higher number of sulfate groups (the “**” group), a slower speed of weight loss than that in sample C was noticed (the arrow in Figure 11), which suggested that the sulfate groups might act as flame retardants, as reported by Roman and Winter [24].

**Figure 12:**
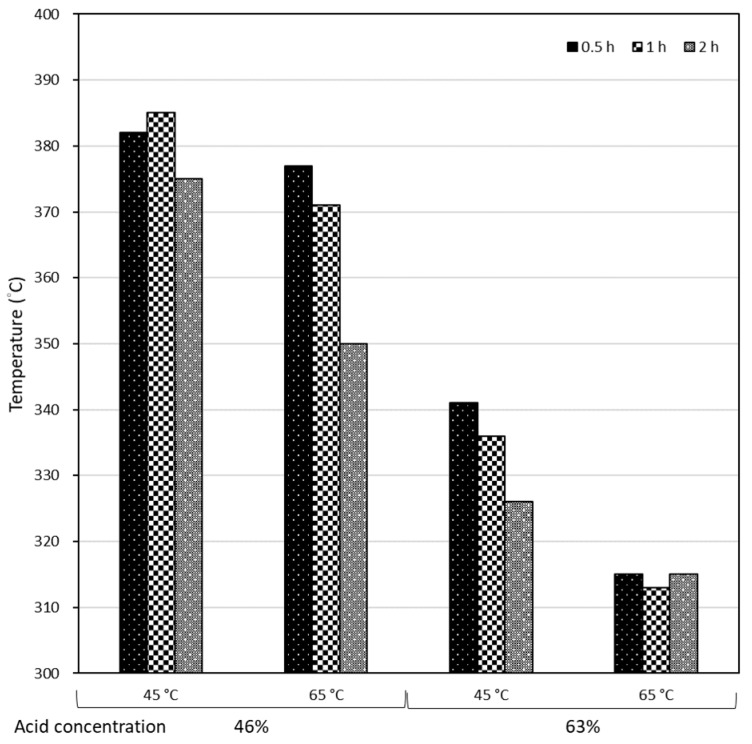
Degradation temperature of P-CNCs prepared in different conditions.

In Figure 12, the thermal degradation temperature decreased with increasing hydrolysis temperature and treatment duration. Accordingly, low hydrolysis temperatures and short treatment durations were suggested in preparing CNCs with a high thermal degradation temperature.

In summary, the resulting P-CNCs revealed an average length shorter than 500 nm for samples prepared at a high sulfuric acid concentration (63%, *w*/*w*). Regarding thermal stability, a lower hydrolysis temperature (45 °C) was preferred to prepare CNCs with a higher thermal degradation temperature. Moreover, the application of a longer hydrolysis duration (2 h) was required to obtain P-CNCs with an optimal *Cr.I.* value. Similar conditions were also chosen in the literature, with the purpose of obtaining an ideal length, crystallinity, as well as an onset temperature of main degradation (Table 2, results of this study are showed in red color). Then, based on the result of “200P” and the reference “Danial- CNC [15]”, hydrolysis duration was suggested to be the parameter that most affected the *Cr.I.* in the case of recycled short fibers.

### 3.4. Surface Morphology

Commercial pulps are known to contain minerals because native ash and tap water washing are used. The mineral impurities might return to insoluble ash, though they should be dissolved during sulfuric acid hydrolysis. The minerals in the dry hardwood pulp contained magnesium, silicon, calcium, chlorine, and potassium, as shown in the resulting diagram from EPMA. Thus, careful pretreatment was deemed a necessary process, even though bleached pulp was chosen.

**Figure 13:**
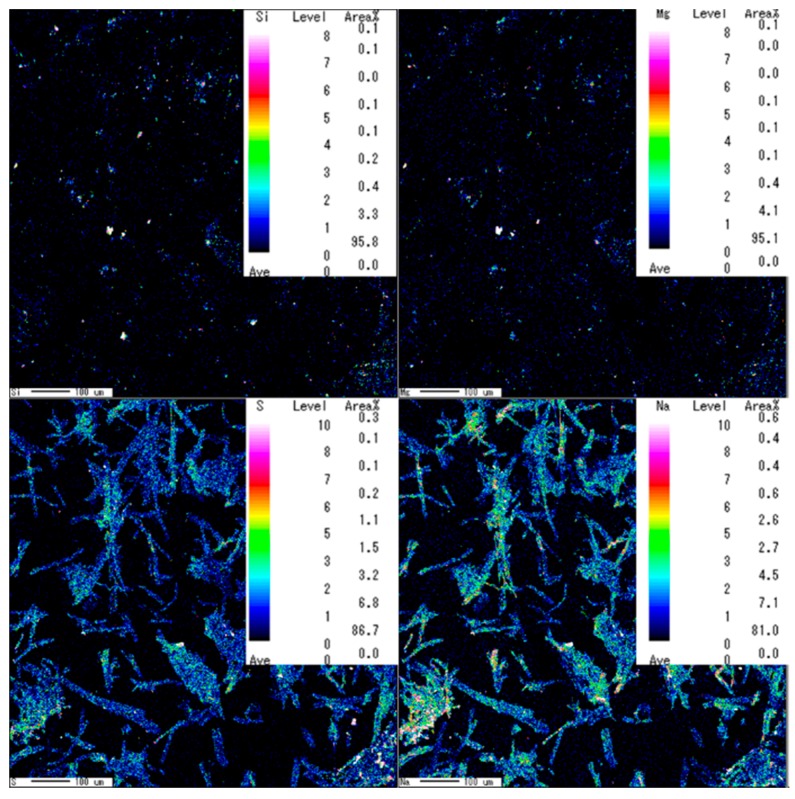
Detection of S and Na in samples: “SA6345-2PN”.

Silica, aluminum, and Magnesium were detected in hardwood pulp and in the sample prepared by 46% sulfuric acid, as suggested by EPMA results. Then, P-CNCs hydrolyzed with a 63% sulfuric acid at 45 °C for 2 h also had remaining magnesium and silicon residues (Figure 13), which meant they were not removed by alkaline extraction nor hydrolysis at such a high sulfuric acid concentration. Less sulfate and sodium remained on the surface of P-CNCs hydrolyzed at 65 °C, as indicated by very weak peaks even under a high-resolution scan. Thus, a high temperature is not recommended for preparing redispersible P-CNCs. However, the P-CNCs hydrolyzed with 46% sulfuric acid showed the presence of other impurities such as calcium.

**Figure 14:**
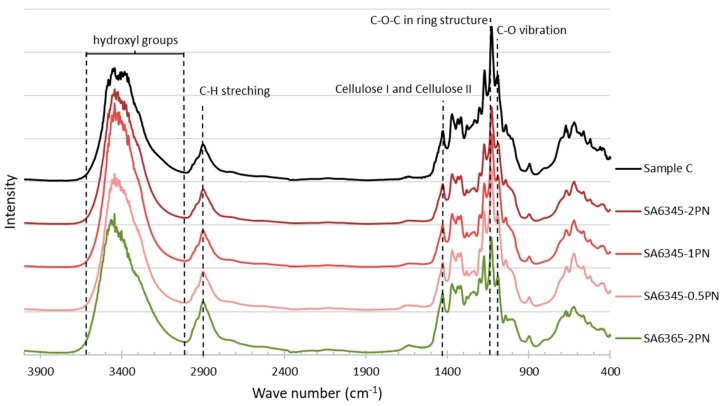
FT-IR spectra of all samples.

Then, surface functional groups were detected by both FT-IR and XPS. In the results of FT-IR, characteristic peaks of cellulose were observed with C–O vibration at 1080 cm^−1^, C–O–C in ring structure at 1130 cm^−1^, and cellulose I and cellulose II at around 1400 cm^−1^. A slightly increased intensity of the C–O–C bond with a decreased hydrolysis temperature was observed because of the removal of the amorphous region (Figure 14), though no significant changes in the FT-IR spectrum suggested no secondary products formed.

**Figure 15:**
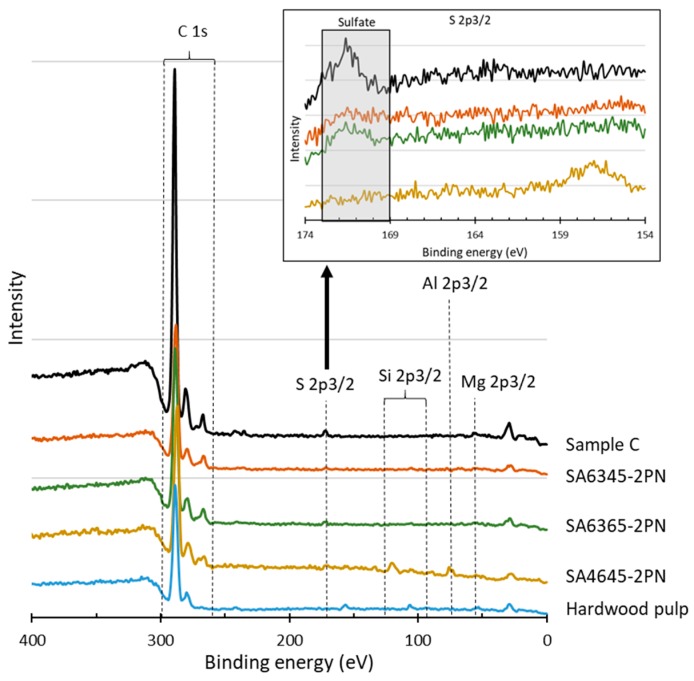
XPS spectrum and magnification of S2p3/2.

**Figure 16:**
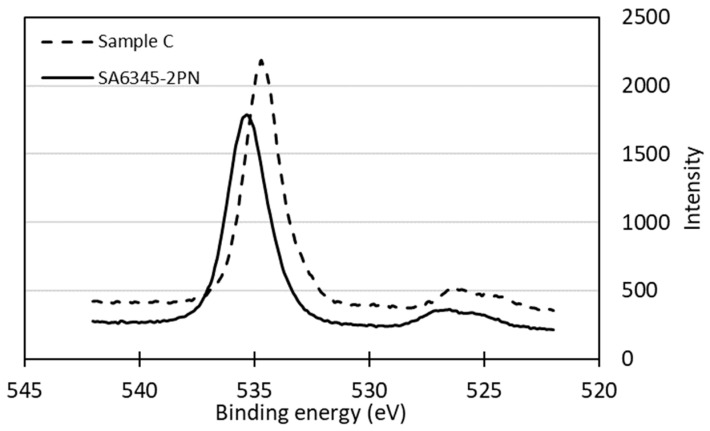
XPS spectrum of O1s in “SA6345-2PN” and sample C.

The sulfate groups introduced via sulfuric acid hydrolyzation were measured at a binding energy around 170 eV by XPS. An increased value of sulfate in SA6365-2PN was observed at a higher acid concentration and hydrolysis temperature (Figure 15). Also, a peak contributed by sodium at around 260 eV was detected in CNC samples and sample C, but not in hardwood pulp (the star mark in Figure 15), which suggested the existence of similar structure of the produced CNCs. However, the O1s peak of P-CNCs (taking SA6345-2PN as an example) shifted to the left, suggesting that P-CNCs contained sodium salts and absorbed more moisture [25,26,27] (Figure 16). However, neither SA6345-2PN nor SA4645-2PN showed an obvious sulfate peak. Instead, a high C/O ratio of SA6345-2PN (0.82) and SA4645-2PN (0.91) was found when it came to overall areas of O1s and C1s XPS signals. Whereas, C/O ratios of 0.78 and 0.61 were calculated for SA6365-2PN and sample C, respectively, which showed a peak of sulfate, indicating the presence of non-cellulosic materials as sulfate groups. Considering the results from both EPMA and XPS, the detected sulfate and sodium was more significant in SA6345-2PN, and most of them were suggested to exist as salts rather than forming sulfate groups on the surface. Not only might the efficiency of composite manufacturing be influenced, but the extra remaining salt might also lead to disadvantages, such as too much moisture absorption. For a longer shelf life and further usage, an improved preparation process is needed to remove most of the salt.

## 4. Conclusions

Dry hardwood pulps were tested for their applicability in CNC fabrication with a variety of hydrolysis conditions, and the resulting products were evaluated. Impurities other than silicon and magnesium were almost fully removed by sulfuric acid hydrolysis, especially with a high acid concentration. However, silicon and magnesium were not removed through alkaline extraction nor acid hydrolysis. Nevertheless, the resulting P-CNCs had an ideal length, thermal stability, and *Cr.I.* at a high sulfuric acid concentration, and they had a lower hydrolysis temperature (45 °C) and a longer hydrolysis duration (2 h). Also, with testing “200P” as a recycled short fiber, a short hydrolysis duration was recommended to maintain its *Cr.I.* value. When introducing sulfate groups on CNCs, a high hydrolysis temperature (65 °C) was preferred to produce redispersible CNCs with successfully grafted sulfate groups.

## Figures and Tables

**Table 1:** Hydrolysis conditions and sample names in P-CNCs.

Sample Name	Concentration (*w*/*w*%)	Temperature (°C)	Duration (h)
SA4645-0.5PN	46	45	0.5
SA4645-1PN	46	45	1
SA4645-2PN	46	45	2
SA4665-0.5PN	46	65	0.5
SA4665-1PN	46	65	1
SA4665-2PN	46	65	2
SA6345-0.5PN	63	45	0.5
SA6345-1PN	63	45	1
SA6345-2PN	63	45	2
SA6365-0.5PN	63	65	0.5
SA6365-1PN	63	65	1
SA6365-2PN	63	65	2

**Table 2:** Effect of hydrolysis conditions on the resulting CNC properties.

Sample	Raw Material	Sulfuric Acid Concentration (%, *w*/*w*)	Hydrolysis Temp. (°C)	Hydrolysis Duration (min)	Yield (%)	Length (nm)	Onset Degradation Temp. (°C)	*Cr.I.* (%)
Sample C	Wood pulp	--	--	--	--	168 ± 3	235	77.0
SA6345-2PN	Hardwood pulp	63	45	120	78.0	224.8 ± 23	263	76.0
SA6345-1PN	Hardwood pulp	63	45	60	79.3	229.2 ± 52	248	73.2
SA6345-0.5PN	Hardwood pulp	63	45	30	77.1	271.1 ± 42	271	72.3
200P	Pre-milled hardwood pulp	63	45	120	29.0	95.2 ± 37	137	47.2
Reid- AITF [9]	Softwood pulp	63	45	120	--	134 ± 56	>250	91.2
Reid- Lab-made [9]	Cotton	64	45	45	--	132 ± 55	>250	93.4
Beck-Candanedo-S2 [20]	Bleached softwood	64	45	45	--	120 ± 5	--	--
Beck-Candanedo-S1 [20]	Bleached softwood	64	45	25	--	141 ± 6	--	--
